# COVID-19 and Ventilation in the Home; Investigating Peoples’ Perceptions and Self-Reported Behaviour (the COVID-19 Rapid Survey of Adherence to Interventions and Responses [CORSAIR] Study)

**DOI:** 10.1177/11786302211015588

**Published:** 2021-05-14

**Authors:** Louise E Smith, Henry WW Potts, Richard Amlȏt, Nicola T Fear, Susan Michie, G James Rubin

**Affiliations:** 1 Institute of Psychiatry, Psychology and Neuroscience, King’s College London, London, UK; 2 NIHR Health Protection Research Unit in Emergency Preparedness and Response, UK; 3 Institute of Health Informatics, University College London, London, UK; 4 Behavioural Science Team, Emergency Response Department Science and Technology, Public Health England, UK; 5 King’s Centre for Military Health Research and the Academic Department of Military Mental Health, King’s College London, London, UK; 6 Centre for Behaviour Change, University College London, London, UK

**Keywords:** COVID-19, ventilation, effectiveness, self-efficacy, confidence

## Abstract

Ventilating indoor spaces helps prevent COVID-19 transmission. We investigated self-reported rates of opening windows to improve ventilation in the home, perceived effectiveness of opening windows, and confidence that if you wanted to, you could open windows. One in 6 people reported rarely, if ever, opening windows in their home in the last week. Three in 4 people knew that opening windows to improve ventilation was an effective way to prevent the spread of COVID-19 and 5 in 6 were confident that they could open windows in their home. Official messaging should continue to seek to improve knowledge about the effectiveness of ventilation for reducing COVID-19 transmission, and increase the frequency of window opening.

COVID-19 spreads through droplet and airborne transmission.^1^ Droplet transmission occurs when an infected individual coughs, sneezes or speaks, releasing droplets into the air that deposit quickly, typically within 2 m. If there is direct air flow from an infected individual, transmission can occur at a greater distance.^2^ Airborne transmission occurs when smaller aerosols carrying a virus evaporate to form droplet nuclei that remain suspended in the air for long periods. Ventilation prevents the spread of infection by diluting droplet nuclei in the air and extracting them outdoors where they are dispersed.^3^ Improving ventilation in indoor spaces reduces transmission of COVID-19.^4^ This is important in home settings, where other protective measures (eg, physical distancing, wearing a face covering) may be less likely, and in non-domestic settings, where there is increased household mixing. Opening doors or windows also has other health benefits by dispersing airborne pollutants. We investigated self-reported rates of opening windows to improve ventilation in the home, perceived effectiveness for opening windows, and confidence that if you wanted to, you could open windows (self-efficacy).

Data from CORSAIR, a series of nationally representative cross-sectional online surveys conducted by BMG Research on behalf of the Department of Health and Social Care, England, were used (collected 26 October to 2 December 2020; n = 10 207 responses from 10 199 participants). This period spanned the launch of a marketing campaign highlighting the importance of ventilation in reducing the spread of COVID-19 in England on 18 November 2020.^5^ We asked participants how often in the last 7 days they had “opened windows to improve ventilation in [their] home.” Participants were asked to what extent they agreed that opening windows regularly to improve ventilation in indoor spaces was an effective way to prevent the spread of COVID-19 and they were confident they could open windows regularly to improve ventilation in their home and other indoor spaces. We investigated whether perceptions and behavior changed over time (comparing survey waves). We coded answers of “not applicable” or “don’t know” as missing (self-reported behavior n = 122, 1·2%; perceived effectiveness n = 216, 2·1%; confidence n = 144, 1·4%). This work was conducted as part of a service evaluation of the marketing and communications run by the Department of Health and Social Care, and so did not require ethical approval.

One in 6 people reported rarely, if ever, opening windows in their home in the last week (Table 1). Only 3 in 4 people agreed that opening windows to improve ventilation was an effective way to prevent the spread of COVID-19 and 5 in 6 were confident they could open windows. There was no difference in self-reported behaviour (χ^2^(20) = 15·0, *P* = .78), perceived effectiveness (χ^2^(20) = 22·3, *P* = .32) or confidence (χ^2^(20) = 22·3, *P* =.32) or confidence (χ^2^(20) = 24·3, P = .23) over time.

**Table 1.** Numbers (n) and percentages (%) of people who reported opening their windows in the last 7 days, and perceived effectiveness of, and confidence for opening windows to improve ventilation.

Response options	Behaviour in last 7 days n = 10 085, n (%)	Response options	Perceived effectiveness n = 9991, n (%)	Confidence n = 10 063, n (%)
Very frequently	3120 (30.9)	Strongly agree	3688 (36.9)	4534 (45.1)
Frequently	2783 (27.6)	Agree	3970 (39.7)	3945 (39.2)
Occasionally	2386 (23.7)	Neither	1703 (17.0)	1120 (11.1)
Rarely	1066 (10.6)	Disagree	394 (3.9)	320 (3.2)
Never	730 (7.2)	Strongly disagree	236 (2.3)	144 (1.4)

Percentages may not sum to 100% due to rounding errors.

Opening windows for short periods of time (10 minutes every hour or 2 hours) may be effective at reducing transmission without compromising temperature. The importance of ventilation in preventing the spread of COVID-19 should be emphasised in official messaging to help improve knowledge about the effectiveness of ventilation for reducing COVID-19 transmission and the frequency of people opening windows at home.
