# Corrigendum to COVID-19 and ventilation in the home; investigating peoples’ perceptions and self-reported behaviour (the COVID-19 Rapid Survey of Adherence to Interventions and Responses [CORSAIR] study)

**DOI:** 10.1177/11786302221109025

**Published:** 2022-06-27

Smith LE, Potts HWW, Amlôt R, Fear NT, Michie S and Rubin GJ. COVID-19 and ventilation in the home; investigating peoples’ perceptions and self-reported behaviour (the COVID-19 Rapid Survey of Adherence to Interventions and Responses [CORSAIR] study) *Environ Health Ins*. 2021: 1-2.

In the above-mentioned article, the authors have noticed some errors. There have been some errors with the datasets delivered to the authors. There was a problem concerning the variable denoting responder ID. For one panel used, all respondents were assigned unique IDs each time they completed a survey, regardless of whether they had previously completed the survey. Therefore, repeat respondents were not appropriately identified. This affected data in waves 8 to 57 (inclusive). The authors have since worked with the market research companies to rectify this problem. The previously published manuscript states that “10,207 responses from 10,199 participants” were used. The corrected figures are 10,207 responses from at least 10,152 participants.

This change does not affect the results nor conclusions in the manuscript.

The online files have been updated.

